# Supplementary material for: PNPLA3 allele frequency has no impact on biliary bile acid composition or disease course in patients with primary sclerosing cholangitis
Source: PLoS One. 2022 Dec 1;17(12):e0277084. doi: 10.1371/journal.pone.0277084 (PMC9714899; doi:10.1371/journal.pone.0277084)
Supplement: Attachment — Submitted filename: Response to reviewers comments 091022.docx [file pone.0277084.s002.docx]

**Response to reviewers’ comments**

Reviewer #1: Thank to the authors who addressed properly to all the raised questions. No further comments from this reviewer

Reviewer #2: In the abstract: I would change “clinical manifestations” with clinical outcomes.

The change has been done and the term ‘clinical manifestations’ has been replaced by ‘clinical outcomes’.

In the conclusions “Conclusions: PNPLA3 I148M variants have no a significant impact on either on bile composition including UDCA content, clinical manifestations, progression of liver fibrosis, the risk for hepatobiliary cancers, transplantation, or overall survival”. Check the grammar. For example on both before and after either and a before significant.

The conclusion in abstract has been rephrased as follow:” Conclusions: The PNPLA3 I148M variant had no significant impact on either on bile composition, including UDCA content, clinical outcomes, progression of liver fibrosis, hepatobiliary cancer risk, liver transplantation, or overall survival.

In the introduction:

- The reviewer would shorten the very long introduction. For example, the sentence “However, PSC is associated with markedly increased risk of cholangiocarcinoma (CCA) (2), with lifetime risk 10%-20% (3,4), or 398 - 1000-fold compared to the general population (4,5). Several risk factors for CCA in patients with PSC, such as inflammatory bowel disease (IBD), especially ulcerative colitis (6), and older age at PSC diagnosis have been identified (4,7).” Is not so important in the context of the manuscript, in fact in the following part of the introduction you mention in patients with PNPLA3 variants have an increased risk of HCC and you don’t mention anymore CCA.

The introduction has been shortened as suggested by reviewer #2: the sentence in rows 50-53 has been deleted. See also below the response to next reviewer’s comment.

The patients with PNPLA3 variants have been reported to have an increased risk of HCC and but there is no published data that the variants would increase the risk for CCA.

Some of the points explained in the introduction should be moved to the discussion which is shorter with respect to the introduction.

The following sentence from introduction (rows 81-87) has been moved into the discussion:” A recent study evaluating plasma BA profiles in PSC to predict hepatic decompensation, demonstrated that the risk was associated with increased concentration of total BA and conjugated fraction of many BA. In contrast, higher glycine: taurine conjugation ratios were protective (24). Intestinal dysbiosis has been described in PSC patients with and without IBD (25). The effect of the PNPLA3 rs738409 variant on gut microbiota has been evaluated only in a few studies. Monga Kravetz A et al. (26) demonstrated that this PNPLA3 variant caused an increased on Firmicutes/Bacteroides-ratio in obese youth with NAFLD.”

Results:

- Wording error in line 192.

The spelling mistake has been corrected.
